# Swallowing a Silver Half-dollar—Death

**Published:** 1883-07-20

**Authors:** 


### THE SWALLOWING OF A SILVER HALF-DOLLAR—DEATH.

A young man about twenty years of age lost a silver half-dollar while throwing it up and catching it in his mouth. He looked for it about the room, but failing to find it, and feeling a sensation in his throat, concluded that he must have swallowed it. On trying to eat he experienced difficulty in swallowing, which was much less after one or two attempts. For several days he ate only soft food, swallowing with difficulty. After that he took his usual diet. He consulted a doctor, who gave him an emetic, and another who twice passed a probang. The latter assured him that the coin must have passed into the stomach, and that the sensation which still persisted was simply the result of irritation.

Nineteen days after the accident he had an attack of hiccough while at dinner. After this the sensation of pressure in the throat was less. He grew pale and thin. He slept little, and could rest only with his head high. He was very nervous, and after about two days complained of feeling very tired. He then had repeated attacks of hæmatemesis, became unconscious, and died twenty-two days after losing the coin.

I give the following notes of the autopsy from the memorandum of Dr. D. S. Burr:

BINGHAMTON, N. Y., July 19, 1879.

Stomach normal, filled with black blood clotted. Upper part of small intestine normal, and filled with black grumous blood. Œsophagus: posterior to commencement of descending aorta the coin was found, with two small perforations into the descending aorta. The coin was a silver half-dollar, and presented the flat sides toward the back and front.

The peculiar features in the case are: First. The point of lodgment. The coin appears to have been moving with sufficient force to pass the constriction at the level of the cricoid cartilage and yet to have been too large to reach the cardiac orifice.

Second. The transverse position in a vertical plane, which, with the backward curve of the œsophagus, might enable the probang to pass in front of it without the operator feeling any obstruction. At any rate it did not present a serious obstacle to the passage of food.

Third. The absence of any extensive inflammation. The only discoverable lesion being the small ulcerations into the aorta, through which the patient slowly bled to death. hemorrhage probably beginning at the attack of hiccough three days before death.—*Boston Med. and Surg. Journal.*

